# Comparison of Immediate, Medium, and Long-Term Postoperative Results of Open Surgery and Fenestrated/Branched Stent Grafts for Extended Thoracoabdominal Aortic Aneurysms

**DOI:** 10.3390/jcm12237207

**Published:** 2023-11-21

**Authors:** Frédéric Cochennec, Thibault Couture, Laurent Chiche

**Affiliations:** Department of Vascular and Endovascular Surgery, Pitié-Salpêtrière Hospital, Assistance Publique Hôpitaux de Paris, Sorbonne Université, 75013 Paris, France

**Keywords:** thoracoabdominal aortic aneurysms, endovascular aortic repair, fenestrated and branched stent grafts, open aortic repair

## Abstract

The optimal surgical treatment for extended thoracoabdominal aortic aneurysms (TAAAs) is still a matter of debate. The historical treatment is open repair (OR), but over the past fifteen years, endovascular strategies have gained widespread acceptance. Although several endovascular techniques have been described for the treatment of TAAAs, fenestrated and branched stent grafts (F/BEVARs) are the most frequently used and best documented. They have become the first-line treatment for both high- and moderate-risk surgical patients in most vascular centers. However, no randomized study comparing OR and F/BEVAR has been published, and decision-making is mainly based on the physician’s preference and/or hospital expertise. The objective of this manuscript is to provide an overview of current comparative data for OR and F/BEVAR.

## 1. Introduction

Extended thoracoabdominal aneurysms (TAAAs) are defined as types I, II, III, and V according to the modified Crawford classification [1]. For vascular surgeons, they represent one of the most challenging pathologies. There is a consensus for surgical repair in low- to moderate-risk surgical patients with intact degenerative TAAAs larger than 60 mm. Repair is reasonable for lower diameters in patients with symptoms or at increased risk of rupture, such as connective tissue disorders, an increase in diameter > 1 cm/year, saccular aneurysm, the presence of a penetrating ulcer, or a significant change in aneurysm appearance [2,3]. 

Despite advances in surgical technique and perioperative critical care, traditional open repair (OR), even in the most experienced centers, remains a major intervention. Severe complications are frequent, including death, paraplegia related to spinal cord ischemia, stroke, renal insufficiency requiring dialysis, respiratory failure, and cardiac complications. As endovascular technology and intraoperative imaging have constantly improved over the past two decades, stent grafts have played a growing role in the treatment of TAAAs. Before totally endovascular approaches became available for TAAAs, hybrid surgery was frequently used in high-risk patients. It consists of safeguarding the perfusion of renal and visceral vessels by means of retrograde bypasses connected to iliac arteries or to the infrarenal aorta before excluding the aneurysms with a stent graft. Hybrid repair has the theoretical advantages of avoiding a thoracotomy and the need for extracorporeal circulation. Although early and mid-term encouraging results generated enthusiasm [4,5], no randomized study comparing OR and hybrid repair has been published. Currently, the hybrid technique is limited to high-risk patients unfit for OR with anatomic contraindications to a purely endovascular treatment. Percutaneous endovascular techniques are increasingly used to treat extended TAAAs. They are mainly represented by custom-made fenestrated and branched stent grafts (F/BEVAR). Other techniques, such as physician-modified stent grafts, in situ fenestrations, and parallel techniques, have been proposed, but they remain off-label. They are generally proposed for high-risk patients who cannot wait for manufacturing delays for a custom-made device because of very large or painful aneurysms. They will not be discussed in the present manuscript. F/BEVAR for TAAAs remains a complex procedure but is now performed on a regular basis in high-volume aortic centers. It has now reached a state of maturity. Recent publications have shown excellent short-term and mid-term results with a high technical success rate and a low rate of postoperative complications. However, long-term data remains limited. Since no randomized study comparing OR and F/BEVAR has been published, the optimal strategy to treat patients with extended TAAAs is still debated. Decision-making is mainly based on the physician’s preference and/or hospital expertise. The objective of this manuscript is to provide an overview of current results and comparative data for these two treatments.

## 2. Results of Open Surgery

Open repair of an extended TAAA typically requires a thoraco-phreno-lumbotomy or a thoraco-phreno-laparotomy. Before the 1990s, the “clamp-and-sew” technique was the primary surgical approach. Surgical techniques and adjuncts have been developed over the years to reduce end-organ and spinal cord ischemia (SCI). They include extracorporeal circulation with distal perfusion and sequential aortic clamping [6,7,8,9,10], selective visceral and renal perfusion with blood or cold crystalloid [11,12,13], hypothermia [14,15,16,17], intercostal artery reimplantation [18,19,20], perioperative monitoring of spinal cord function using motor-evoked potentials and somatosensory-evoked potentials [21,22,23,24], and cerebrospinal fluid drainage [25,26].

### 2.1. Early Results

Most publications reporting on elective open TAAA repair are monocentric, with a retrospective design. They originate from high-volume centers [14,17,27,28,29,30,31,32,33,34,35,36,37,38,39]. In these series, in-hospital mortality varies from 5 to 15%, major adverse events from 15 to 45%, spinal cord ischemia from 5 to 15%, stroke from 5 to 10%, and renal failure from 3 to 30%. Respiratory failure is the most frequent complication, occurring in up to 60% of cases. Gastrointestinal complications might be underestimated in already-published reports. In a recent series of 3587 open TAAA repairs, they occurred in 5.9% of cases [40]. Complication rates vary according to the proportion of patients treated emergently, the proportion of extended TAAAs (Type I, II, and III TAAAs vs. Type IV), and comorbidities.

Outside these high-volume centers, mortality and complication rates increase significantly. A US state-wide study reported 30-day and 1-year mortality rates after elective open TAAA repair of 19% and 31%, respectively [41]. Other population-based studies have shown similar results [42,43]. It is now well documented that postoperative mortality correlates with surgeon and hospital volume [42,44]. These results suggest that OR of extended TAAAs should be performed only in high-volume aortic centers. 

Several meta-analyses and systematic reviews reporting on open TAAA repair have been published [45,46,47]. A summary of the early outcomes reported in these studies is given in Table 1. Results should be taken with caution since they included historical series published before the evolution of operative strategies and the use of adjuncts significantly improved outcomes [48]. They may not reflect the current results of OR in high-volume centers. The definition of outcomes such as SCI and renal complications was inconsistent between studies. Most studies did not describe the extent and duration of neurologic injury. They reported heterogeneous results with heterogeneous techniques. Another meta-analysis focused on SCI after open and endovascular repair of descending thoracic aneurysms and TAAAs [49]. The pooled rate of permanent SCI was 7% after open TAAA repair.

### 2.2. Long-Term Results

In the long term, there is a substantial amount of data showing that open TAAA repair is a durable treatment with acceptable rates of aortic reinterventions and branch instability. In a recent Canadian population-based study from Rocha et al., secondary vascular procedures were necessary in 13.5% of patients at eight years [50]. In the largest single-center series of open TAAA repair from Coselli et al., freedom from repair failure, defined as freedom from graft infection, pseudoaneurysm formation, or patch aneurysm, was 97.9% at 5 years and 94.1% at 15 years [27]. The most frequent causes of reintervention were graft infection and pseudoaneurysm formation. Another large single-center retrospective study reported excellent long-term patency of visceral and renal vessel reconstructions: estimated 5-year patencies of the celiac, SMA, left renal, and right renal were 99%, 100%, 97%, and 96%, respectively [51]. 

Despite good and durable technical results, there are data suggesting that even if a patient has been successfully discharged from the hospital, the patient after open TAAA repair is unlikely to return to the life expectancy of the general population with the same demographic characteristics [52]. The population-based study from Rocha et al. showed that after open TAAA repair, overall survival rates at 1 and 8 years were 77.8% and 48.1% [50]. Long-term data from expert centers showed similar results: Coselli et al. [27] reported an 83% survival rate at one year and a 64% survival rate at five years. In another large single-center series, survival rates were 54.4% at 5 years, 28.7% at 10 years, and 20.5% at 15 years [53]. Several studies have identified variables that have been associated with mortality over time. In addition to older age and acute presentation, other factors such as preoperative chronic kidney disease, peripheral artery disease, hypertension, and congestive heart failure have been identified as negative factors for long-term survival [52,54]. Aneurysm extent has been clearly associated with postoperative mortality and SCI rates, with type II TAAAs being associated with the highest risks. Whether aneurysm extent affects the long-term outcome after open TAAA repair remains unclear. 

In addition to mortality, reinterventions, and visceral vessel patency, other endpoints should be considered to evaluate long-term results. TAAA life-altering events (TALEs) have been recently proposed as a composite endpoint to evaluate more accurately the long-term disability caused by the intervention. It includes mortality, permanent paraplegia, permanent dialysis, and stroke. In the population-based study from Rocha et al., freedom from TALE at 1 and 8 years was 69.5% and 39.9% [50]. In other words, eight years after TAAA open repair, only 39.9% of patients were alive and free from permanent paraplegia, stroke, or dialysis. 

Overall, open TAAA repair is a major operation. Low rates of postoperative mortality and acceptable rates of severe complications have been reported by high-volume centers. OR is a durable treatment, but reinterventions for graft infection, pseudoaneurysm formation, and target vessel instability may be required in some patients during follow-up. Despite good and durable technical results, long-term freedom from mortality and freedom from TALE are low. 

### 2.3. Chronic Dissections

Because of the presence of the intimal flap, the OR of post-dissection TAAAs is often considered more complex than the repair of degenerative aneurysms. They are more extensive, develop in younger patients, and typically require longer visceral and spinal cord ischemia times. An early report from a high-volume center suggested that chronic dissection was a risk factor for SCI [55]. With surgical adjuncts, postoperative complications and SCI rates have reduced significantly, and whether the technical complexity of the OR of post-dissection TAAAs currently has an impact on postoperative outcomes remains unclear. One recent retrospective study analyzed the effect of chronic dissection on the postoperative outcomes of open TAAA repair [56]. Among 453 patients who underwent OR for type I to III TAAA, 90 had a chronic dissection. Patients with chronic dissection had higher rates of perioperative mortality (12% vs. 6%) and major adverse events (59% vs. 42%) when compared with degenerative aneurysms. There was also a trend towards higher rates of SCI (18% vs. 12%), but this difference did not reach statistical significance. Age-adjusted long-term survival was not different between the two cohorts. Other studies have found that chronic dissection is not associated with negative outcomes after open TAAA repair [54,57,58].

### 2.4. Connective Tissue Disorders

OR remains the gold standard for patients with connective tissue disorders. The recent guidelines on aortic disease from the American College of Cardiology/American Heart Association have given a class I recommendation for open TAAA repair in patients with connective tissue disorders such as Marfan syndrome, Loeys–Dietz syndrome, or vascular Ehlers–Danlos syndrome [3]. Although numerous studies have reported outcomes of open TAAA repair in the general population, data on patients with connective tissue disorders are limited. Historical data suggested similar mortality rates but higher rates of SCI in patients with Marfan syndrome [59]. More recent series have reported more favorable outcomes, with operative mortality, SCI, and stroke rates not exceeding 5% [35,60,61,62,63,64,65,66,67]. Early reintervention for bleeding is not rare, occurring in up to 10% of patients. These series reported good long-term survival rates (>80% at five years and >75% at ten years), better than those usually reported in older patients with degenerative disease. Depending on prior aortic surgery and the extent of replacement, the need for reoperation during follow-up for proximal growth, distal growth, patch aneurysm, or valve replacement varies among series, occurring in up to 30% of cases [60]. LeMaire et al. reviewed their experience with 178 open TAAA repairs in patients with Marfan syndrome or suspected Marfan syndrome [68]. They reported excellent short-term and long-term results. Perioperative mortality, SCI, stroke, and dialysis reoperation for bleeding occurred in 3%, 4%, 1%, 3%, and 3%, respectively. At five years, survival was 83% and freedom from repair failure was 98%. Lau et al. [69] recently published their results of 684 open TAAA repairs in patients with (N = 90) or without (N = 594) Marfan syndrome [69]. In patients with Marfan syndrome, operative mortality (3.3%), major adverse events (13.3%), SCI (3.3%), and dialysis (3.3%) were not statistically different from patients without connective tissue disorders. Survival at ten years in Marfan patients was 78.7%. The cumulative risk for reoperation was higher in Marfan patients (14.7% vs. 3.4%). One of the largest series of open TAAA repairs in patients with heritable aortic disease comes from the Genetically Triggered Thoracic Aneurysms and Cardiovascular Conditions (GenTAC) Registry [61]. The data of 142 patients were examined (76 with Marfan syndrome, 31 with familial thoracic aortic aneurysms and dissections, 10 with Loeys–Dietz syndrome). The operative mortality rate was 1.3%. Paraplegia occurred in 4% of cases, and acute renal failure in 5%. The most common complication was vocal cord paralysis (21%). 

## 3. Results of Endovascular Techniques

Fenestrated and branched stent grafting for extended TAAAs is technically challenging and requires specific imaging technology and endovascular skills. Visceral and renal vascularization is insured by custom-made fenestrations, outer branches, or inner branches. The off-the-shelf T-branch Cook device has been developed to treat symptomatic patients who cannot wait for custom-made stent graft manufacturing. It is not widely available yet. The choice between branches and fenestration depends on anatomical characteristics and the surgeon’s preference. Branches are preferentially planned for large aortic diameters when the graft is not against the aortic wall and when the target arteries have a downward orientation. Fenestrations are preferred when the stent graft body is against or close to the aortic wall or when the target arteries have an upward orientation. F/BEVAR was initially limited to high-risk patients, but over the last 15 years, after encouraging short-term and mid-term results were published, it has gained popularity for low-/moderate-risk patients. Constant technical improvement finally led F/BEVAR to a state of maturity. Although F/BEVAR may reduce postoperative complications, SCI remains a concern. Similar to OR, several surgical adjuncts have been developed over time to reduce the incidence of SCI. They include CSF drainage, procedure staging, segmental artery coil embolization, early reperfusion of internal iliac arteries, preservation of aortic collaterals such as the left subclavian artery, blood pressure optimization, and blood transfusion [70]. Early reports advocated the use of prophylactic CSF drainage, but this strategy has been challenged by recent studies [71,72] due to substantial risks of complications, moderate rates of SCI, and frequent improvement after therapeutic CSF drainage [73,74,75]. 

### 3.1. Early Results

Like OR, the results of F/BEVAR mainly originate from high-volume centers. Recently, efforts have been made in Europe and the US to create multicenter registries, such as the United States Aortic Resarch Consortium (US_ARC), the SVS Vascular Quality Initiative registry, or the International Aortic Research Consortium.

In large retrospective studies including more than 100 patients [70,74,75,76,77,78,79,80,81,82], technical success is >95%. Perioperative mortality varies from 3% to 9%, SCI from 3% to 15%, stroke from 1% to 3%, and acute kidney injury from 3% to 10%. Major adverse events are recorded in 20–40% of cases. All these studies showed that, in contrast to OR, the majority of neurologic deficits were incomplete and recovered, with a majority of patients regaining ambulatory function [83]. Permanent paraplegia did not exceed 5% of cases. 

A recent study extracted from the SVS Vascular Quality Initiative Registry the data of 1139 patients who underwent endovascular extended TAAA repairs [84]. Postoperative mortality and TALE rates were 5.9% and 10%, respectively. Aneurysm extension and pre-existing chronic kidney disease were associated with the occurrence of TALE. Data from the International Aortic Research Consortium showed that staging elective F/BEVAR of extended TAAA was associated with a lower risk of mortality and/or permanent paraplegia and with higher survival at 1 and 3 years [85].

A recent meta-analysis included 2333 patients with TAAAs from 27 studies [86]. The pooled rate of mortality was 7%. In the subgroup of patients with extended TAAAs, the pooled SCI rate reached 13%. Staging the procedure was again found to significantly reduce the rate of SCI. 

Whether aneurysm extent has a real impact on outcome after F/BEVAR for TAAAs is still debated. A large population-based study demonstrated that aneurysm extent had a negative impact on outcomes [87]. In contrast, a multicenter registry from expert centers suggested that the outcomes of extensive and nonextensive TAAAs were similar [83].

### 3.2. Long-Term Results

In the mid-term (<4 years), F/BEVAR for extended TAAAs is well documented and has shown excellent freedom from aortic-related mortality rates. However, the need for reinterventions for persisting endoleaks and target vessel instability remains a concern. Target vessel instability after F/BEVAR is a composite endpoint defined as any branch-related complication leading to aneurysm rupture, death, occlusion, component separation, or reintervention to maintain branch patency or to treat a branch-related component separation or endoleaks. In the long term, data are still limited, and studies with a mean follow-up period longer than 30 months are scarce. 

In one of the largest series of F/BEVAR for extended TAAAs (n = 354) in high-risk patients [76], freedom from aneurysm-related death at 3 years was 91%, freedom from all-cause mortality was 57%, and freedom from unplanned secondary intervention was 54%. These reinterventions were mainly performed for endoleaks or to maintain branch vessel patency. Of note, this study included patients between 2004 and 2013, a period during which the technique had not reached a state of maturity. In other large retrospective studies, reinterventions were needed in 20–50% of cases, depending on follow-up duration [77,78,79,88]. They were mostly endovascular and rarely the cause of death from severe complications [89]. 

Data on the mid-term fate of target and visceral vessels have been reported by multicentric studies [90,91,92,93,94,95]. Estimated rates at 5 years of freedom from target artery instability have been evaluated at around 85–90%. These studies suggested higher rates of target vessel instability with branches compared with fenestrations and with balloon-expandable bridging stents compared with self-expandable stents. 

### 3.3. Chronic Dissections

Even in high-volume aortic centers, the clinical experience with F/BEVAR for chronic post-dissection TAAAs remains limited. Recently published multicenter studies have given additional insights, suggesting that F/BEVAR is safe and provides encouraging short-term and mid-term results. Again, longer follow-up is needed to better evaluate the role of endovascular techniques in the treatment of chronic post-dissection TAAAs. 

A multicenter international study analyzed the outcomes of F/BEVAR in 246 patients treated for chronic post-dissection extended TAAAs [96]. Technical success was 96%. Postoperative mortality was low (3%) but major adverse events were frequent, occurring in 28% of cases. However, disabling complications were uncommon: rates of permanent paraplegia, stroke, and new-onset dialysis were 2%, 1%, and 1%, respectively. Survival estimates were in line with previous series of the OR of chronic post-dissection extended TAAAs: 79% at 3 years and 65% at 5 years. Freedom from aortic-related mortality was 95% at 3 years and 93% at 5 years. Freedom from secondary interventions at 5 years was 44%, higher than rates usually reported in OR series. These reinterventions were mainly related to target vessel instability. Another multicenter cohort study showed similar short-term results [97]. During follow-up, when compared with patients with degenerative TAAAs, endoleaks were more frequent in post-dissection patients, but overall survival and freedom from aorta-related deaths were similar. This is consistent with the findings of a recent retrospective study showing higher rates of target artery-related endoleaks in chronic dissections compared with degenerative TAAAs [98]. 

### 3.4. Connective Tissue Disease

Data on F/BEVAR for TAAAs related to connective tissue disorders are still lacking. Only a few studies with limited numbers have been published [99,100,101]. In a recent literature review [102], 26 patients with TAAAs related to connective tissue disorders and treated by F/BEVAR could be identified. Early results were excellent, with no perioperative mortality and no SCI, but long-term results were not reported. Until additional long-term data are available, F/BEVAR for extended TAAAs in patients with connective tissue disorders seems reasonable in high-risk patients when stent grafts can be deployed in previously implanted synthetic grafts.

## 4. Open Repair vs. F/BEVAR for TAAAs: Results of Comparative Studies

No randomized study comparing OR and F/BEVAR for extended TAAAs has been published. As most vascular surgeons have developed specific skills and preferences for one technique or the other, such a study is unlikely to be conducted. Comparative data are mainly based on cohort retrospective studies or nationwide data. In most of these studies, patients were treated before 2015, a period during which F/BEVAR for extended TAAA was not mature. They included heterogeneous patients with heterogeneous aetiologies and various aneurysmal extents, including type IV TAAAs. 

### 4.1. Clinical Outcomes

The largest single-center comparative retrospective study included 1053 patients with extended TAAAs (457 OR, 596 endovascular) [103]. After propensity score matching, in-hospital mortality (OR: 8.3% vs. endovascular: 7.6%), paraplegia, and stroke (3.6% vs. 2.2%) rates were similar between the two groups. Dialysis-dependent acute renal failures were more frequent in the OR group (8.6% vs. 3.3%). In matched patients, OR resulted in better 10-year survival (53% vs. 33%) and less frequent aortic reintervention (4% vs. 21%). In contrast, other single-center experiences with limited numbers suggested reduced early mortality and complication rates after F/BEVAR, but no clear difference could be demonstrated for SCI [104,105].

Nation- or state-wide comparative studies have shown improved outcomes in terms of postoperative mortality and complication rates after F/BEVAR [43,50,52,106]. However, they provided conflicting results for SCI rates. In the long term, F/BEVAR was associated with increased rates of reinterventions, but the type of treatment does not seem to influence long-term survival, which remains poor after open or endovascular TAAA repair. These studies also showed that endovascular repairs became more frequent over time, reaching 74% in 2014 in a German study [43]. 

In their meta-analysis, Rocha et al. analyzed eight comparative studies [107]. The analysis performed on the basis of propensity-matched studies and the analysis performed on the basis of unadjusted studies yielded conflicting results in terms of postoperative mortality and SCI. These studies were estimated to be at high risk of bias. The same group conducted another systematic review that included 71 studies reporting outcomes of OR or F/BEVAR in at least 10 patients with TAAAs [47]. This study failed to show a difference in terms of perioperative mortality (OR: 8.9% vs. F/BEVAR: 7.4%), but patients were older with more comorbidities in the endovascular cohort. OR was associated with lower rates of SCI (10.5% vs. 13.5%), but rates of permanent paralysis were similar (6.7% vs. 5.2%). Postoperative dialysis was more frequent in the OR group (12.0% vs. 6.4%), but rates of permanent dialysis at discharge were similar (3.8% vs. 3.7%). A more recent meta-analysis found reduced mortality, cardiac, and respiratory complication rates in patients treated with F/BEVAR [108]. However, the type of treatment did not seem to influence the incidence of paraplegia. 

Overall, there is a reasonable amount of data showing that F/BEVAR is associated with reduced early mortality and complication rates. Whether F/BEVAR is able to reduce SCI and permanent paraplegia is unclear. However, when SCI occurs, recovery seems to be more frequent after F/BEVAR compared to OR [83]. In the long term, comparative data are too scarce to draw any firm conclusion. 

### 4.2. Cost-Effectiveness Studies

Several studies compared the cost-effectiveness ratio of OR vs. endovascular techniques for the treatment of TAAAs. They provided conflicting results. In a recent US nationwide study including 879 intact TAAAs treated between 2009 and 2015, the adjusted total hospitalization cost was significantly higher after OR (*p* = 0.005) [109]. In this study, the in-hospital cost was three times higher after OR. This higher cost was probably explained by a higher length of stay. In the French WINDOW registry, costs related to endovascular treatment were not significantly different from those related to OR for TAAAs at 30 days and 2 years [106,110]. In another nationwide study from Canada, costs were higher at 1 month after endovascular repair (CAD 62,802 vs. CAD 33,605; *p* < 0.01), but there was no difference 12 months after repair [111]. 

### 4.3. Chronic Dissections

One recent meta-analysis compared OR and F/BEVAR for post-dissection TAAAs: 1337 patients (1068 in the OR group and 269 in the F/BEVAR group) from 15 cohort studies were included in the analysis. No significant difference was found in terms of early mortality (OR: 6% vs. endovascular: 3%), SCI (OR: 9% vs. endovascular: 8%), or early reintervention rate (OR: 3% vs. endovascular: 1%). The only significant difference was the rate of pulmonary complications, which was higher in the OR group (OR: 30% vs. endovascular: 2%). During follow-up, no significant difference could be identified between overall mortality and aortic-related mortality. Late aortic reinterventions were more frequent after endovascular repair (OR: 11% vs. endovascular: 32%) [112].

## 5. Conclusions

In the absence of a randomized study, the level of evidence showing that endovascular techniques are able to improve the outcome of TAAAs is low. Recent data suggest that F/BEVAR is able to reduce early mortality and complication rates compared to OR. Regarding spinal cord ischemia, comparative studies have provided conflicting results. Aortic reinterventions during follow-up are more frequent after F/BEVAR, especially in patients with chronic dissention. Most of them are related to endoleaks or target artery instability. They are most often endovascular and rarely the cause of severe complications. In the long term, comparative data are too scarce to draw a firm conclusion. Open repair remains a valid and durable option, especially in young patients and patients with chronic dissections and connective tissue disorders. 

## Figures and Tables

**Table 1:** Open TAAA repair: results of systematic reviews and meta-analysis.

Study	Number of Patients/Number of Analyzed Studies	Pooled Perioperative Mortality Rates	Pooled Spinal Cord Injury Rates	Pooled Stroke Rates	Pooled Renal Complication Rates	Pooled Respiratory Complication Rates	Pooled Cardiac Complication Rates	Early Reoperation for Bleeding
Khan et al. [45]	12,245/54 No volume cutoff for inclusion	All types: 10.5% Type I: 10.5% Type II: 15.2% Type III: 13.1% Type V: 13.7%	Permanent: 6.0% Temporary: 4.3%	5.3%	renal failure: 16.6%	26.8%	2.7% (MI)	NR
Moulakakis et al. [46]	9963/30 cutoff point of 50 patients for inclusion	All types: 11.3% Type I: 7% Type II: 10.3% Type III: 8.0%	Paraplegia: 5.0% Paraparesis: 3.6%	3.1%	Permanent dialysis: 7.9%	23%	4.4%	Varied from 1.5% to 13.1%
Rocha et al. [47]	NR/47 Cutoff point of 10 patients for inclusion	All types: 8.9%	SCI events: 7.4% Permanent paraplegia: 4.4%	3.9%	Acute renal failure: 21.7% Postoperative dialysis: 12% Permanent dialysis: 3.8%	NR	NR	Pooled proportion: 5.3%

MI: myocardial infarction; NR: not reported; SCI: spinal cord ischemia.

## Data Availability

Not applicable.
